# Pediatric COVID-19: Correlations between Clinical and Imaging Perspectives

**DOI:** 10.1155/2023/4159651

**Published:** 2023-05-03

**Authors:** Heba A. Ali, Shaimaa A. Mohammad

**Affiliations:** ^1^Department of Pediatrics, Pulmonology Division, Faculty of Medicine, Ain Shams University Children's Hospital, Cairo, Egypt; ^2^Department of Diagnostic and Interventional Radiology and Molecular Imaging, Faculty of Medicine, Ain Shams University Hospital, Cairo, Egypt

## Abstract

**Background:**

Although SARS-CoV-2 infection primarily affects adults, the increasing emergence of infected pediatric patients has been recently reported. However, there is a paucity of data regarding the value of imaging in relation to the clinical severity of this pandemic emergency.

**Objectives:**

To demonstrate the relationships between clinical and radiological COVID-19 findings and to determine the most effective standardized pediatric clinical and imaging strategies predicting the disease severity. *Patients and Methods*. This observational study enrolled eighty pediatric patients with confirmed COVID-19 infection. The studied patients were categorized according to the disease severity and the presence of comorbidities. Patients' clinical findings, chest X-ray, and CT imaging results were analyzed. Patients' evaluations using several clinical and radiological severity scores were recorded. The relations between clinical and radiological severities were examined.

**Results:**

Significant associations were found between severe-to-critical illness and abnormal radiological findings (*p* = 0.009). In addition, chest X-ray score, chest CT severity score, and rapid evaluation of anamnesis, PO2, imaging disease, and dyspnea-COVID (RAPID-COVID) score were significantly higher among patients with severe infection (*p* < 0.001, <0.001, and 0.001) and those with comorbidities (*p* = 0.005, 0.002, and <0.001).

**Conclusions:**

Chest imaging of pediatric patients with COVID-19 infection may be of value during the evaluation of severe cases of infected pediatric patients and in those with underlying comorbid conditions, especially during the early stage of infection. Moreover, the combined use of specific clinical and radiological COVID-19 scores are likely to be a successful measure of the extent of disease severity.

## 1. Introduction

Coronavirus disease 2019 (COVID-19) is a respiratory tract infection caused by a newly emergent coronavirus, SARS-CoV-2. The pathogen caused an outbreak of respiratory illness that rapidly spread worldwide and was accepted by the World Health Organization (WHO) as a pandemic disease [1]. Nowadays, it is considered a global public health emergency [2]. Initial studies suggest that the immune system plays a detrimental role in the pathogenesis of coronavirus infection [3, 4].

Pediatric patients account for approximately 2% of the reported cases [5, 6], and nearly 0.9% of all affected patients are younger than 15 years of age [7]. Several studies suggest that children are not severely affected by coronaviruses as commonly as the adult population because of differences in the function of the immune system, but rather, they may act as silent carriers [8]. In addition, a small percentage of pediatric patients may develop severe disease or lose their lives [9].

A confirmed diagnosis of COVID-19 infection requires PCR identification of viral nucleic acid [10]. Furthermore, previous studies suggest that computed tomography (CT) chest imaging may have a higher sensitivity and prognostic value [11]. It may be considered an effective method for the detection of lung abnormalities, especially in the early stages of the disease among symptomatic adult patients [12]. However, the role of radiologic examinations in initial diagnosis, disease progression evaluation, and prognosis is currently a topic of active research and discussion in the pediatric community [13]. CT findings in children have been described in small cohorts only [14, 15], making efficient diagnosis challenging.

When making imaging decisions in pediatric patients suspected of having COVID-19 infection, multiple factors should be considered, including the sensitivity and specificity of radiologic examinations, the availability and accuracy of reverse transcription-polymerase chain reaction (RT-PCR) tests, and radiation dose considerations. Many pediatric patients will eventually undergo some level of imaging evaluation. However, there is currently little information available describing the imaging manifestations of COVID-19 in pediatric patients and even less information discussing the use of imaging studies in pediatric patients. As the pandemic spreads, practical guidance for pediatric radiologists and referring physicians is required to address both of these concerns [13].

Furthermore, there is limited data at present about the relationship between radiological findings indicative of COVID-19 severity and clinical presentation in pediatric patients with coronavirus infection. Therefore, the aim of this study was to describe the clinical characteristics and the key imaging features of COVID-19 in the pediatric population in order to determine the most precise standardized pediatric clinical and imaging approaches predicting disease severity.

## 2. Subjects and Methods

### 2.1. Study Design and Settings

This observational retrospective study was conducted at a single tertiary center. The study was reviewed and approved by the Research Ethical Committee, Faculty of Medicine, in our institute. We analyzed 80 consecutive patients with SARS-CoV-2 infection who were admitted to our institute during the period between January 1, 2021, and March 1, 2022. The institute has been declared to be a specialized isolation center for managing pediatric patients with COVID-19 infection during the epidemic period.

### 2.2. Study Subjects

All the studied patients aged less than 18 years were confirmed to have COVID-19 infection through a positive reverse transcription-polymerase chain reaction (RT-PCR) assay of respiratory secretions from the nasopharynx and oropharynx for COVID-19 nucleic acid [16–18]. PCR testing was performed on the same day of admission. Patients with unavailable or negative RT-PCR testing were omitted from the study.

### 2.3. Data Collection

Sociodemographic data, including age, gender, medical history, contact history, clinical features, underlying comorbidities, physical examination findings, time of onset of symptoms, and the presence of complications, such as the multisystem inflammatory syndrome in children (MIS-C), were collected. All the patients underwent vital data assessment and arterial blood gas (ABG) analysis, in addition to routine laboratory tests and electrocardiogram (ECG). Radiological investigations, including chest X-ray (CXR), and CT chest findings were also recorded. All patients underwent chest imaging within 3 days after the initial RT-PCR testing. The clinical presentation, laboratory results, and imaging findings were assessed.

### 2.4. Clinical Analysis

According to WHO guidelines [18], all 80 cases were classified into four severity groups: mild, moderate, severe, and critical. The criteria were as follows: (a) Mild cases have no or light symptoms without evidence of hypoxia or viral pneumonia. (b) In the moderate group, cases had fever and respiratory symptoms with no signs of severe pneumonia, including blood oxygen saturation levels (SpO_2_) ≥ 90% on room air. (c) Patients in the severe group have clinical signs of severe pneumonia, plus any one of the following items: respiratory rate > 30 breaths/minute, severe respiratory distress, SpO_2_ < 90% at room air, or signs of pneumonia with a general danger sign, inability to breastfeed or drink, lethargy, unconsciousness, or convulsions. (d) In the critically ill group, patients had any one of the following items: respiratory failure–required mechanical ventilation; shock; and admission to the intensive care unit (ICU) for acute respiratory distress syndrome (ARDS), sepsis, or life-threatening organ dysfunction. Patients also were classified according to the presence or absence of comorbidities. In addition, the COVID-19 severity assessment score (COSA) was calculated. It is a clinical score to predict the likelihood of severe disease courses for SARS-CoV-2-positive patients [19].

### 2.5. Imaging Analysis

The low-dose mode, automatic tube current modulation with a voltage of 120 kVp, matrix size of 512 × 512, and increments and thickness of 1.5–2 mm were used for imaging acquisition as recommended by the thoracic CT protocols [20] using a fixed device devoted for suspected or confirmed COVID-19 cases. CT images were viewed in the transverse, sagittal, and coronal planes. CXR and chest CT images were evaluated at the time of admission using the picture archiving and communication system (PACS) for radiological findings suggestive of COVID-19 infection in accordance with the recommendations of the Radiological Society of North America (RSNA) [21], focusing on the imaging findings with the best resolution. Contrast studies were used only to assess complicated pneumonia, vascular abnormalities, or mediastinal lymphadenopathy. Radiological assessment was performed by a pediatric radiologist who had at least 5 years of experience in pediatric chest imaging and a board-certified pediatric pulmonologist.

Interpretation of imaging findings was done without previous familiarity of a pediatric radiologist with the PCR results. CXR images were assessed with the calculation of the CXR score [22]. In addition, CT images were evaluated by using the recommendations of the nomenclature committee of the Fleischner Society [23]. The presence of mediastinal lymphadenopathy and pleural effusion was documented as well, and the chest CT severity score (CT-SS) was also calculated as previously published [24, 25].

Finally, RAPID-COVID score (rapid evaluation of anamnesis, PO2, imaging disease, and dyspnea-COVID score) was calculated. It is a clinical-radiological index applied to grade the severity of the disease and is based on clinical symptoms and PaO_2_/FiO_2_ and the CXR score [2]. A more detailed description of the COVID-19 severity scores is displayed in the Supplementary data.

### 2.6. Statistical Analysis

All statistical analyses were performed using SPSS V.23.0. Data with numeric variables and normal distributions were presented as means ± standard deviations (SDs) and analyzed using Student's *t* test; nonparametric data were expressed as medians and interquartile ranges (IQRs) and analyzed using the Kruskal-Wallis test or the Mann-Whitney *U* test. Different patients' groups were compared regarding the prevalence of radiological findings and the calculated clinical and imaging scores. Correlations were determined using Spearman's rank correlation coefficient to assess the associations of quantitative data. Categorical variables were analyzed using a chi-square test. A *p* value below 0.05 was considered statistically significant, and *p* less than 0.01 was considered highly significant. The clinical data and the prevalence of radiological findings were described as frequency rates and percentages. Consistency between the severity of lung involvement on chest CT and clinical classification was assessed using the kappa test. Receiver operating characteristic (ROC) analysis was performed to determine the optimal cut-off values of COVID-19 severity scores for discriminating severe from mild to moderate illness. Area under the curve (AUC), sensitivity, and specificity were reported.

## 3. Results

### 3.1. Presenting Characteristics

The analysis was based on 80 pediatric patients (44 males and 36 females), with a median age of 6 years, and ranged from 2 months to 15 years. Sixty (75%) of the patients developed symptoms before hospitalization. Meanwhile, 20 (25%) of the patients developed COVID-19 infection after hospitalization due to another reason. In addition, 40 patients (50%) had an underlying disease, of which immunological diseases represented the most common etiology (30%) (Table 1).

### 3.2. Clinical Features and Disease Severity

The most common presentations reported at the onset of illness were fatigue and fever which were present in 75 patients (93.8%) and 73 patients (91.3%), respectively. Severe-to-critical cases were reported in 57.5% of the studied patients. In addition, multisystem inflammatory syndrome in the children (MIS-C) was present in 30.5% of the patients. The characteristics of the studied patients with MIS-C are summarized in E-Table 1. The median RAPID-COVID score and COSA score were 4 (IQR: 1 to 6) and 3 (IQR: 1 to 5), respectively. The basal sociodemographic and clinical features of pediatric patients with COVID-19 infection are displayed in Table 1.

### 3.3. Imaging Findings and Radiological Severity Scores

**Figure 1:**
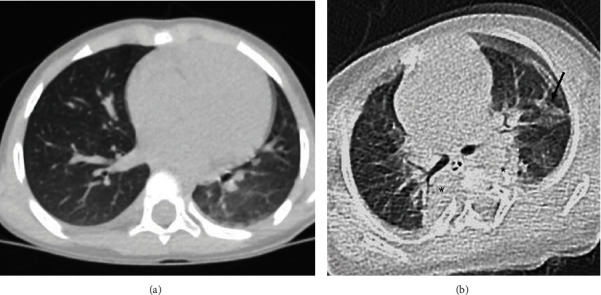
Axial CT of the chest of two different patients with positive COVID-19 RT-PCR test. (a) A 4-year-old female was presented with fever and cough. CT shows a ground-glass opacity involving the left lower lobe, which is an indeterminate chest radiographic finding of pediatric COVID-19 pneumonia. The patient clinically improved and was discharged after 10 days. (b) A 7-month-old female with biliary atresia was presented with fever and respiratory distress. CT shows bilateral patches of consolidation (asterisk) associated with a ground-glass opacity (arrow), which are typical chest radiographic findings of pediatric COVID-19 pneumonia. The patient developed progressive shortness of breath and was transferred to the intensive care unit, was mechanically ventilated, and died.

**Figure 2:**
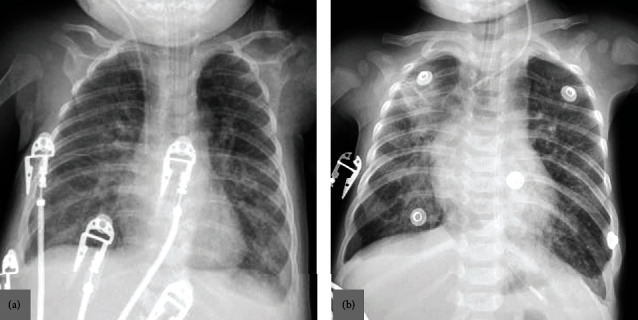
Chest X-rays of two different patients with positive COVID-19 RT-PCR test. (a) A 4-month-old male with a neurometabolic disorder was presented with fever and dyspnea. Chest X-ray shows an ill-defined ground-glass opacity as well as reticular areas of increased opacity involving almost all lung zones on both sides, which is an indeterminate chest radiographic finding of pediatric COVID-19 pneumonia. Also noted are the endotracheal tube and a central line. The patient developed severe hypoxemia, was transferred to the intensive care unit, was mechanically ventilated, and died. (b) A 4-month-old male with Down syndrome was presented with fever and respiratory distress. Chest X-ray shows an ill-defined area of consolidation and ground-glass opacity in the right upper and lower lung zones along with ill-defined diffuse reticular areas of increased opacity in both lungs, which is an indeterminate chest radiographic finding of pediatric COVID-19 pneumonia. Also noted are the endotracheal tube and a central line The patient developed progressive respiratory compromise and was transferred to the intensive care unit, was mechanically ventilated, and died.

**Figure 3:**
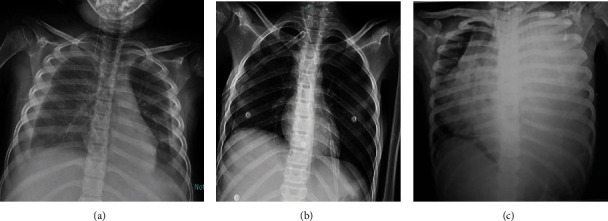
Chest X-rays of three patients with COVID-19 having pleural disease which is an atypical finding for pediatric COVID-19 infection that required intervention. (a) A 5-year-old female with a history of fever and cough. Chest X-ray shows bilateral pleural effusion. The patient was discharged after 12 days. (b) A 14-year-old female with associated tuberculous enteritis and tuberculous lymphadenitis was presented with fever and acute shortness of breath. Chest X-ray shows bilateral pneumothoraces; no parenchymal lung abnormality was detected on CT (not shown). The intercostal tube is also noted. The patient severely deteriorated and was transferred to the intensive care unit, was mechanically ventilated, and died. (c) A 5-year-old female was presented with fever, cough, and respiratory distress. Chest X-ray shows a homogenous opacity involving the left hemithorax associated with a marked mediastinal shift to the contralateral side in keeping with the left-sided pleural effusion. The patient underwent intercostal tube insertion, clinically improved, and was discharged after 15 days.

**Figure 4:**
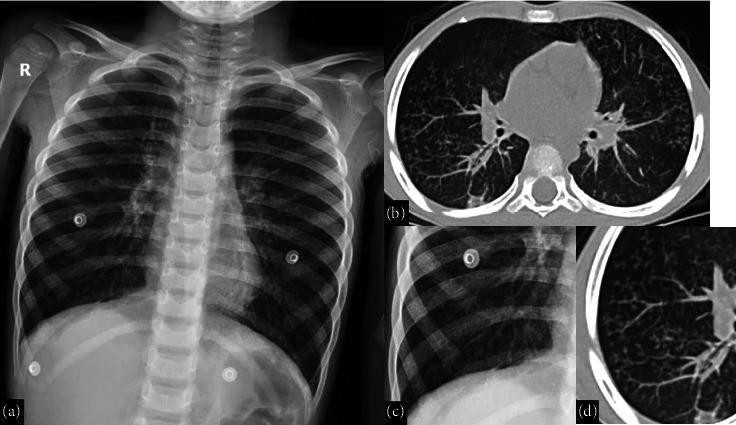
A 6-year-old male with a history of bronchial asthma who acquired COVID-19 infection. He was presented with fever and cough. (a) Chest X-ray with a magnified view (c) shows faint reticulonodular opacities. (b) Axial CT of the chest (lung window) with a magnified view (d) shows centrilobular nodules with opacified distal bronchioles (tree-in-bud sign), which is an atypical finding for pediatric COVID-19 pneumonia. The patient clinically improved and was discharged after one week.

CT of the chest was found to be abnormal in only 45 patients (56.2%). The most common radiological abnormalities were diffuse consolidation (19, 42.2%), ground-glass opacities (GGOs) (15, 33.3%), bilateral peribronchial thickenings and peribronchial opacities (9, 20%), and mixed peripheral GGOs and consolidation (7, 15.6%), while only 5 patients (11.1%) had bilateral peripheral subpleural GGOs in the lower lobes. The typical appearance of COVID-19 based on the RSNA expert consensus statement was detected in only 8.8% of the cases. The key radiological features of the study population have been illustrated in Figures 1–4.

As regards the distribution of radiological lesions in different age groups, ground-glass opacities were significantly more prevalent among infants (46.6%), while consolidation was higher among the scholar age group (31.4%) compared to other age groups (*p* = 0.040).

Concerning the radiological scores, the median CXR score and CT-SS were 2 (IQR = 0 to 4) and 6.5 (IQR = 0 to 15), respectively. Detailed chest-imaging examination results and quantitative CT parameters are given in E-Table 2.

### 3.4. Correlations between COVID-19 Radiological Findings and Clinical Severity

Significant associations were found between abnormal CXR findings and clinical severity (*p* = 0.009). In addition, ground-glass opacities and lung consolidations were specifically observed among patients with critical COVID-19 infection (66.7%) (*p* = 0.024). Moreover, in comparison to CXR radiographic findings, CT chest-imaging features were significantly related to the clinical classification of COVID-19, whereby 27.8% of the critical group had typical radiological findings of lesions for COVID-19 compared to 7.1%, 0.0%, and 0.0% among severe, moderate, and mild groups, respectively (*p* = 0.043). Furthermore, atypical radiological findings were significantly observed among eleven patients (47.2%) with severe and critical illness of whom only four patients had coincident complicated bacterial pneumonia, while the rest had pure COVID-19 illness (Table 2).

Regarding the severity scores, there was a positive relationship between the COSA score and the symptom severity, which was significant only between the mild and moderate groups (*p* = 0.017), while it was significantly elevated among COVID-19 patients complicated with MIS-C (*p* = 0.014) as shown in E-Table 1. Also, there were significant associations between the RAPID-COVID score, CXR score, CT-SS, and severe illness, where higher scores of 6 (1–8), 4 (1–6), and 24 (12–28) were frequently observed among patients with severe-to-critical COVID-19 infection compared to less severe groups (*p* = 0.001, <0.001, and <0.001, respectively). Significant correlations between clinical severity with the shape and the density of the COVID-19-related imaging findings, and severity scores are shown in Table 2. Additional correlations between the COVID-19 severity scores and the other study parameters are presented in E-Table 3.

Furthermore, cut-off values of the COVID-19 severity scores were determined via ROC curve analysis to predict severe cases. It was found that the best diagnostic scores to distinguish severe from mild-to-moderate cases were the RAPID-COVID score with a threshold > 4, AUC = 0.710, sensitivity of 50.00, and specificity of 94.12, followed by CT-SS with a threshold > 9, AUC = 0.656, sensitivity of 58.70, and specificity of 73.53, and CXR score with a threshold > 3, AUC = 0.618, sensitivity of 67.31, and specificity of 53.57 as shown in E-Figure 1.

### 3.5. Relationship between Underlying Comorbidities and Disease Severity

The studied patients with underlying comorbid conditions were more likely to have more severe symptoms (*p* < 0.001), a higher need for intensive care unit (ICU) admission (*p* < 0.001), and mechanical ventilation (*p* < 0.001) due to COVID-19-related illness, compared to those without. Moreover, abnormal radiological findings (*p* = 0.005), higher CXR score (*p* = 0.005), CT-SS (*p* = 0.002), and RAPID-COVID score (*p* < 0.001) were significantly associated with the presence of comorbidities. The clinical and radiological characteristics of the studied patients with underlying comorbid conditions are presented in Table 3.

### 3.6. Relationship between the Radiological Severity and the Disease Outcomes

Regarding the study outcomes, more than half of the patients (56.3%) were admitted to the intensive care unit, and of those, 35% were mechanically ventilated. The need for supplemental oxygen was reported among 43.8% of the studied patients. Of the studied patients, 16.3% died throughout the study, while complicated COVID-19 was observed in 59 (73.75%) of the studied patients. Moreover, higher CXR score and CT-SS were significantly associated with the need for ICU admission (*p* = 0.005 and 0.002), supplemental oxygen (*p* = 0.001 and <0.001), mechanical ventilation (*p* = 0.002 and <0.001), COVID-19 complications (*p* = 0.001 and 0.001), and mortality (*p* = 0.001 and <0.001), as illustrated in the E-Figure 2 and 3 panels.

## 4. Discussion

In the early days of the COVID-19 infection pandemic, pediatric cases were infrequent. Nowadays, the number of children suffering from COVID-19 infection is gradually increasing [15]. However, there are still no adequate structured policies for evaluating COVID-19-positive children. Therefore, the current study was conducted to determine the most efficient and applicable clinical and radiological diagnostic strategies predicting the disease severity, unique for pediatric patients with COVID-19 infection that can be easily used by practitioners and pediatric radiologists for the early identification of COVID-19 patients at risk for severe outcomes, monitoring the disease progression and decreasing the risk of complications. In the current study, we have evaluated the clinical and radiological characteristics of the children and adolescents with COVID-19 infection using different severity scores. This study highlighted that combined clinical and radiological scores appear to be effective for grading COVID-19 severity among pediatric patients and might be recommended for monitoring the patients with severe-to-critical illness.

The present study included 80 pediatric patients with a male-to-female ratio of 1.2 : 1. Their age ranged from 2 months to 15 years, with about half of the patients aged more than 6 years. Associated comorbidities were observed among 50% of the study population. In addition, this work revealed that 20 (25%) of the studied patients developed health care–associated COVID-19 infection which necessitates proper implementation of intensive infection-control strategies in hospital settings to reduce the risk of potential viral transmission to ameliorate the disease severity and alleviate the burden on the health care system.

Regarding the clinical severity of the studied patients, more than half of the patients had severe-to-critical illness with considerable percentages of ICU admission, mechanical ventilation, and mortality. Similar to our results, the CDC [6] stated that although children have milder symptoms overall, severe cases do occur in the pediatric population, and deaths have been reported.

On the other hand, a recent study conducted by Steinberger et al. [26] on thirty pediatric patients with COVID-19 reported that the condition of all patients remained stable throughout hospitalization. No patients required supplemental oxygen, intubation, or ICU admission. In addition, Epidemiology Working Group for NCIP Epidemic Response and Chinese Center for Disease Control and Prevention [27] reported that only 3% of children had severe illness. Consistent with these findings, several published studies [5, 6, 14, 15, 28–30] reported that milder and less prominent clinical symptoms were reported in pediatric patients, with a recovery period of 1–2 weeks, which is shorter than in adult cases [31].

While mild disease forms have been reported to be common in children with COVID-19, our study reported several cases of varying degrees of severity. This may be attributed to being a tertiary care center receiving more cases with comorbidities which highlights that severe illness is not uncommon among COVID-19-infected children as previously reported, which necessitates the urgent need for a pediatric-specific severity-scoring system for the early identification of high-risk patients susceptible to severe illness to limit disease progression.

Our results found that 43.7% of the patients had normal CT findings. COVID-19 pneumonia was reported in 39% of the patients. In addition, 45.0% had bilateral lung involvement. The lower lobes were the most commonly affected areas (81.3%), while 8.9% of those patients had single-lobe involvement. A positive halo sign was observed in 2.2% of the patients in our study.

Our findings are consistent with Palabiyik and colleagues [32], who found that bilateral pneumonia and multifocal pneumonia were the most frequently reported radiological abnormalities, while single-lobe involvement was observed in 27% of the cases. In addition, 69% of the lesions were observed in the lower lobes, and the most common involvement was in the left lower lobe. Furthermore, our imaging findings and their distribution are in agreement with findings published in the literature on COVID-19 in both pediatric and adult populations [11, 14, 33].

In our analysis, it is worth mentioning that the presence of normal radiological findings among 43.8% of the studied patients, who mostly had mild-to-moderate illnesses, indicates that chest imaging may not be necessary and might increase the risk of radiation exposure among these groups of patients.

Similar to other studies [34, 35], we have found that that consolidation was the most common CT finding, followed by GGOs and mixed GGOs and consolidation, while a ground-glass pattern with or without consolidation was the most predominant feature in other reports [15, 26, 32, 34, 36, 37]. GGOs followed by consolidation are the characteristic radiological findings that have been associated with COVID-19 in adults as well [11, 12, 33].

The difference between our study and previous studies may be explained by the diversity of the age groups between the different studies. In addition, most of our study population had severe involvement.

Based on the aforementioned findings, increasing the awareness of pediatric radiologists and clinicians about the key radiological findings, especially atypical ones enclosing bilateral peribronchial cuffing and opacities, bilateral and unilateral segmental consolidation, pleural effusion, and pneumothorax, is recommended.

In the current study, it was observed that radiological lesions were distinct in the different age groups, with a higher prevalence of consolidation in the older children, which was inconsistent with the results of Bayramoglu et al. [38] who reported that no statistically significant differences were found regarding chest radiography and chest CT findings based on the different age groups, which may be due to the smaller numbers of the studied patients.

In this work, abnormal radiological findings were significantly observed among severe and critical cases. In addition, radiological severity scores were significantly associated with symptom severity (Table 2). Moreover, the combined clinical-radiological RAPID-COVID score was the most effective score to predict the disease severity among the studied pediatric patients, with the highest specificity and AUC compared to the other isolated radiological scores (i.e., CXR score and CT-SS).

Ultimately, routine imaging studies are not recommended for pediatric patients with mild-to-moderate symptoms but may be considered in pediatric patients with severe-to-critical illness to monitor clinical deterioration, exclude complications, and detect possible atypical radiological findings that that may necessitate immediate interventions beyond the usual supportive medical therapy, as with other viral respiratory infections [13, 39].

Our findings are consistent with previous reports on cases of COVID-19 infection, which showed that patients in the severe group are more likely to have a higher CT involvement score [40, 41]. Furthermore, Ai et al. reported that in the early period, chest CT findings appeared to correlate with clinical findings without RT-PCR positivity [42]. In addition, Li et al. [43] and Sun et al. [44] stated that high CT scores were features of COVID-19 children with severe or critical status.

Contradictory to our findings, a study conducted by Palabiyik and colleagues [32] on 59 children with COVID-19 pneumonia revealed no correlation between COVID-19 pneumonia and clinical and radiological scores, while Chung et al. [11] and Steinberger et al. [26] reported that no significant association has been established between imaging severity in children and clinical symptoms, which may be due to the small number of patients studied.

In this work, the median CXR score and CT-SS were 2 (IQR = 0 to 4) and 6.5 (IQR = 0 to 15), respectively.

These results are parallel with those of Chung et al. [11], who reported that the mean CT severity score was 6.5. However, our findings were relatively lower than previous studies [45, 46], conducted in adults with confirmed COVID-19 infection, which stated that the mean CT-SS was 11.2 and 13, which indicates a lower threshold of the CT severity score and less pulmonary involvement among the pediatric population compared to adults.

Compared to our study, a recent study conducted by Steinberger et al. [26] on pediatric patients with COVID-19 revealed that the CT-SS ranged from 0 to a maximum of 7, with a mean severity score of 0.7.

In the present study, correlation analysis (E-Table 3) indicated that the RAPID-COVID score, CXR score, and CT-SS had significantly negative correlations with oxygen saturation and positive correlations with the frequency of lobe involvement and respiratory rate. In addition, higher values of inflammatory markers were significantly observed among patients with higher scores which are consistent with previously published studies [47].

Our study found that the typical appearance of COVID-19, according to the RSNA statement, was detected in only 8.8% of the cases (Table 2). Our findings are much lower than in previous studies [38], in spite of the prevalence of severe cases in the study population. This discrepancy may be explained by the lower sensitivity of the RSNA statement in the radiological diagnosis of COVID-19 infection in the study population, as well as the inconsistency between radiological severity as measured by the RSNA statement and the clinical severity in the pediatric age group, which spotlights the need for a precise imaging consensus unique to children including the key radiological findings in the pediatric population, especially the atypical ones which may not be uncommon in this group of patients and might be misinterpreted as other viral infections.

Of note, our study revealed that the CXR score is positively correlated with CT-SS (*p* < 0.001) (E-Table 3). In addition, the value of CXR was enhanced with the addition of clinical parameters, as in the RAPID-COVID score which was found to have the highest AUC compared to the other scores (CT-SS and CXR score). Our results may justify the use of CXR instead of chest CT as a screening tool for children with severe COVID-19 infection to decrease the risk of exposure to ionized radiation in this patient population that has increased radiation sensitivity, which also may be a cost-effective measure especially in communities with restricted resources.

Moreover, the study highlighted that patients with comorbidities may be at higher risk of acquiring severe COVID-19 infection and elevated COVID-19 radiological severity scores, which coincide with a recent study conducted by Aimen et al., who reported that COVID-19 patients with underlying comorbid conditions are at risk for prolonged hospital stay and a poor outcome [48]. These findings highlighted that the patients with coexisting medical conditions might be in need of routine PCR screening; should be treated with a high index of suspicion, especially when symptomatic; and should undergo close follow-up using clinical and radiological COVID-19 scores if severe symptoms develop.

In addition, it was noted that the studied patients with complicated COVID-19 were more likely to have higher radiological severity scores which may reveal the value of chest imaging in verifying the emergence of complications in poorly responsive patients with severe disease. Similarly, higher severity scores were significantly associated with the need for ICU admission and mechanical ventilation compared to those with better outcomes, which may highlight the prognostic value of chest imaging for anticipation of severe patients at risk for poor outcomes, which may affect the treatment strategy for early aggressive intervention and close follow-up to decrease the risk of complications, especially if atypical radiological findings could be encountered.

To the best of our knowledge, this is one of the fewest studies that addressed different radiological features among the children and adolescents with COVID-19 infection, demonstrating their parallel relationship with symptom severity in the study population. In addition, the study may reveal the value of chest imaging in categorizing the COVID-19 patients with severe illness in need for advanced health care facilities, frequent monitoring, and intensive management to preserve medical resources.

Based on our results, it is worth mentioning the necessity of implementing a simple, reliable, and optimal combined clinical and radiological algorithm unique for pediatric patients with COVID-19 infection, for the overall assessment of all aspects of the disease and early identification of severe COVID-19 cases, thus reducing the utilization of intensive care facilities.

There were some limitations to this study. First, this was a single-center study, so further longitudinal multicentric studies with longer follow-up measures are needed to confirm the efficacy of these clinical and radiological approaches in predicting the clinical outcome, monitoring disease progression, and evaluating treatment response. Second, higher percentages of COVID-19 patients with comorbid conditions were included in the study, so further studies on patients with isolated COVID-19 disease may be needed to address the peculiar effect of COVID-19 infection; however, considerable numbers of COVID-19 pediatric patients who need intensive medical care have comorbidities, which necessitate further studies. Third, unhospitalized patients were not included in the study, as they usually have mild symptoms which may need no further intervention. Finally, chest imaging may not be indicated in all cases of the studied population.

In conclusion, our study revealed that imaging findings of pediatric COVID-19 may speculate on clinical severity. However, the value of chest imaging may be limited to COVID-19 patients with severe illness and those with comorbidities, which may have a clinical impact and might affect the management strategy and help in triaging the patients in need for advanced health care facilities. In addition, its usefulness may be enhanced with proper clinical assessment, and so, COVID-19 infection might be managed same as other viral cases of pneumonia.

## Figures and Tables

**Table 1:** Demographics and clinical characteristics among the studied COVID-19 patients.

Variables (*n* = 80)	
Age in years, median (IQR)	6 (2–10)
Range	2 months–15years
Males (%)	44 (55.0%)
Onset of the disease	
Before hospital admission	60 (75%)
Hospital acquired	20 (25%)
Duration of the disease (days), mean ± SD	13.48 ± 5.79
Range	4–30
Fatigue	75 (93.8%)
Fever (%)	73 (91.3%)
Respiratory manifestations	72 (90%)
Upper respiratory tract symptoms	72 (90%)
Lower respiratory tract symptoms	47 (58.8%)
Wheezes	50 (62.5%)
Dyspnea	47 (58.8%)
Sore throat	26 (32.5%)
Rhinorrhea	8 (10.0%)
Anosmia	1 (1.3%)
Oxygen saturation	94.45 ± 2.03
Skin manifestations (%)	35 (43.8%)
Cardiac manifestations (%)	28 (35.0%)
Myalgia and bony aches	25 (31.3%)
Headache and irritability	16 (20.0%)
Neurological manifestations (%)	14 (17.5%)
Complicated COVID-19 (%)	59 (73.75%)
Pneumonia	23 (39%)
Pleural effusion	7 (11.8)
MIS-C	18 (30.5%)
Septic shock	9 (15.3%)
Encephalitis	2 (3.4%)
Comorbid conditions (%)	40 (50%)
Immunological diseases	12 (30%)
Surgical problems	7 (17.5%)
Chronic respiratory diseases	4 (10.0%)
Malignancy	4 (10.0%)
Chronic renal diseases	4 (10.0%)
Neurological disorders	3 (7.5%)
Hematological diseases	2 (5.0%)
Diabetic ketoacidosis	2 (5.0%)
Biliary atresia	1 (2.5%)
Chromosomal disorders	1 (2.5%)

MIS-C: Multisystem inflammatory syndrome in children.

**Table 2:** Relations between COVID-19 clinical classification, radiological findings, and severity scores.

Variables	Clinical classification of COVID-19				*P* value
	Mild	Moderate	Severe	Critical	
	No. = 12	No. = 22	No. = 28	No. = 18	
Abnormal chest X-ray findings	4 (33.3%)	9 (40.9%)	16 (57.1%)	16 (88.9%)	0.009^∗∗^
Ground-glass opacities and lung consolidations	1 (8.3%)	8 (36.3%)	11 (39.3%)	12 (66.7%)	0.024^∗^
Peribronchial thickenings and peribronchial opacities	3 (25%)	3 (22.7%)	5 (17.8%)	2 (11.1%)	0.961
Pleural effusions	0 (0.0%)	2 (9.0%)	4 (14.3%)	1 (5.6%)	0.834
Pneumothorax	0 (0.0%)	1 (4.5%)	0 (0.0%)	2 (11.1%)	0.559
Cardiomegaly	0 (0.0%)	0 (0.0%)	2 (7.1%)	2 (11.1%)	0.361
RSNA chest radiographic finding suggestive for COVID-19					
Negative	10 (83.3%)	10 (40.9%)	12 (42.8%)	3 (16.7%)	
Indeterminant	1 (16.7%)	11 (50.0%)	8 (28.6%)	7 (38.9%)	0.060
Typical	0 (0.0%)	0 (0.0%)	2 (7.2%)	4 (22.2%)	
Atypical	1 (8.3%)	1 (4.5%)	6 (21.4%)	4 (22.2%)	
RSNA CT chest findings suggestive for COVID-19					
Negative	10 (83.3%)	10 (40.9%)	12 (42.9%)	3 (16.7%)	
Indeterminant	1 (16.7%)	10 (45.5%)	7 (25%)	6 (33.3%)	0.043^∗^
Typical	0 (0.0%)	0 (0.0%)	2 (7.1%)	5 (27.8%)	
Atypical	1 (8.3%)	2 (9%)	7 (25%)	4 (22.2%)	
COVID-19 severity assessment score, median (IQR)	0 (0–4)	4.5 (2–5)	3 (2–5)	3.5 (2–5)	0.017^∗^
RAPID-COVID score, median (IQR)	1 (1–4)	2 (1–6)	3 (1–6)	6 (1–8)	0.001^∗∗^
Respiratory failure	0 (0.0%)	0 (0.0%)	6 (21.4%)	16 (88.9%)	0.001^∗∗^
Chest X-ray score, median (IQR)	0 (0–6)	1 (0–6)	2 (1–6)	4 (1–6)	<0.001^∗∗^
Chest CT severity score (CT-SS), median (IQR)	0 (0–4)	6 (0–12)	6 (0–14)	24 (12–28)	<0.001^∗∗^

RSNA: Radiological Society of North America Expert Consensus Statement; RAPID-COVID score: rapid evaluation of anamnesis, PO2, imaging disease, and dyspnea-COVID score. ^∗^*p* is significant; ^∗∗^*p* is highly significant.

**Table 3:** Comparison between the clinical and radiological characteristics of the studied patients with and without comorbidities.

Variables	COVID-19 patients without comorbidities	COVID-19 patients with comorbidities	
	No. = 40	No. = 40	
Wheezes	18 (45.0%)	32 (80.0%)	<0.001^∗∗^
Respiratory distress	15 (37.5%)	32 (80.0%)	0.001^∗∗^
Respiratory rate, mean ± SD	29.29 ± 9.73	35.89 ± 11.35	
Range	16–55	16–55	0.008^∗∗^
Cardiac manifestations	6 (15.0%)	22 (55.0%)	0.003^∗∗^
Clinical severity			
Mild	9 (22.5%)	3 (7.5%)	
Moderate	19 (47.5%)	3 (7.5%)	
Severe	10 (25.0%)	18 (45.0%)	<0.001^∗∗^
Critical	2 (5.0%)	16 (40.0%)	
Intensive care unit admission	10 (25.0%)	35 (87.5%)	<0.001^∗∗^
Mechanical ventilation	1 (2.5%)	27 (67.5%)	<0.001^∗∗^
Mortality	1 (2.5%)	12 (30.0%)	0.004^∗∗^
COVID-19 severity assessment score			
Median (IQR)	3 (0–5)	4 (2–5)	0.212
Range	0–6	0–6	
Abnormal radiological findings	16 (40.0%)	29 (72.5%)	0.005^∗∗^
Lesion size and density			0.029^∗^
Ground-glass opacities (GGOs)	7 (17.5%)	8 (20.0%)	
Consolidation	3 (7.5%)	16 (40.0%)	
Ground-glass opacities (GGOs) and consolidation	3 (7.5%)	4 (10.0%)	
Bronchiectasis changes	1 (2.5%)	0 (0.0%)	
Number of lobes affected			0.018^∗^
Median (IQR)	0 (0–3)	3 (0–4)	
Range	0–6	0–6	
The affected lung sides			0.024^∗^
No	26 (65.0%)	9 (22.5%)	
Bilateral	10 (25.0%)	26 (65.0%)	
Unilateral	4 (10.0%)	5 (12.5%)	
RSNA expert consensus statement			0.046^∗^
Negative	26 (65.0%)	9 (22.5%)	
Indeterminate	10 (25.0%)	14 (35.0%)	
Atypical	4 (10.0%)	10 (25.0%)	
Typical	0 (0.0%)	7 (17.5%)	
Chest X-ray score			0.005^∗∗^
Median (IQR)	0 (0–3)	4 (0–4)	
Range	0–6	0–6	
RAPID-COVID score			<0.001^∗∗^
Median (IQR)	1 (1–2)	5 (2–6)	
Range	1–6	1–8	
Chest CT severity score (CT-SS)			0.002^∗∗^
Median (IQR)	0 (0–10)	12 (0–24)	
Range	0–32	0–32	

RSNA: Radiological Society of North America Expert Consensus Statement. ^∗^*p* is significant; ^∗∗^*p* is highly significant.

## Data Availability

All data generated or analyzed during this study are included in this published article (and its supplementary information files).

## Supplementary Materials

Supplementary Materials Supplementary data: detailed description of the severity scores used among the studied COVID-19 patients. Supplementary tables: E-Table 1: comparison between the basic characteristics of the studied patients with and without MIS-C. E-Table 2: detailed radiological findings among the studied patients with COVID-19 infection. E-Table 3: correlations between COVID-19 severity scores and the other study parameters. Supplementary figures: E-Figure 1: ROC curves to predict the cut-off values of the different COVID-19 severity scores. E-Figure 2 panel: relations between chest X-ray score, intensive care unit (ICU) admission (2A), mechanical ventilation (2B), complications (2C), and mortality (2D). E-Figure 3 panel: relations between chest CT severity score (CT-SS), intensive care unit (ICU) admission (3A), mechanical ventilation (3B), complications (3C), and mortality (3D).
