# Supplementary material for: Pediatric COVID-19: Correlations between Clinical and Imaging Perspectives
Source: Pulm Med. 2023 May 3;2023:4159651. doi: 10.1155/2023/4159651 (PMC10171977; doi:10.1155/2023/4159651)

**Pediatric COVID-19: Correlations Between Clinical and Imaging Perspectives**

**Online Supporting Information**

1. **Supplementary data:**

COVID 19 severity scores:

1. Chest X-ray (CXR) score: It is a radiological score, aimed at evaluating Covid-19 manifestations in the lung (22). This CXR scoring system includes two steps of imaging analysis. on frontal chest projection (PA or AP), marked as letters A, B, and C for the right lung and D, E, and F for the left lung. The letters divide lungs into three levels: upper level (A and D) – above the inferior wall of the aortic arch; middle level (B and E) – below the inferior wall of the aortic arch and above the inferior wall of the right inferior pulmonary vein (the hilar structures); and lower level (C and F) – below the inferior wall of the right inferior pulmonary vein (the lung bases) .The second step is to assign a score (from 0 to 3 points) to each zone based on the detected lung abnormalities: 0 – no lung abnormalities; 1 – interstitial infiltrates; 2 – interstitial and alveolar infiltrates (interstitial predominance); and 3 – interstitial and alveolar infiltrates (alveolar predominance). The overall score is the sum of the points from all the zones with a range from 0 to 18.
2. CT severity score (CT-SS): The CT-SS is used to describe ground-glass opacity, interstitial opacity, and air trapping. According to the anatomic structure, the 18 segments of both lungs were divided into 20 regions, in which the posterior apical segment of the left upper lobe was subdivided into apical and posterior segmental regions, whereas the anteromedial basal segment of the left lower lobe was subdivided into anterior and basal segmental regions. The lung opacities in all of the 20 lung regions were evaluated on chest CT images using a system attributing scores of 0, 1, and 2 if parenchymal opacification involved 0%, less than 50%, or equal to or more than 50% of each region, respectively. The CT-SS was defined as the sum of the individual scores in the 20 lung segment regions, which may range from 0 to 40 points (24,25).
3. RAPID-Covid score (Rapid evaluation of Anamnesis, PO2, Imaging disease, Dyspnea-Covid Score), a clinical-radiological index applied to grade the severity of the disease and based on clinical symptoms, PaO2/FiO2 and the CXR score. The RAPID-Covid score ranges from 1 to 10 points, assigned as follows: Presence of symptoms as fever, cough, gastrointestinal symptoms =1point; dyspnea =2points; PaO2/FiO2 ratio: >350=0point; 350-300=2points; <300=4points - CXR score: 0points if CXR score ≤ 3; 2points if CXR score 4 or 5; 4points if CXR score ≥6 (2).
4. COVID‑19 severity assessment (COSA) score: It is a clinical score that accurately predicted the likelihood of severe disease courses for SARS-CoV-2 positive patients. The most predictive risk factors were male sex, low hemoglobin (< 100 g/L), elevation of inflammatory parameters (CRP > 25 mg/L, leucocyte counts > 10 G/L), hyperglycemia (> 10 mmol/L), and impaired renal function (eGFR < 75 mL/min, sodium > 144 mmol/L), where higher values indicating a higher risk for worse outcomes, a total score of up to 3 was associated a low risk. A score of 4 or 5 was associated with a moderate risk, while 6 to 7 points were associated with high risk, and 8 or more points correlated with a very high risk to develop a severe COVID-19 (19).
5. **Supplementary Tables:**

E-Table 1: Comparison between the basic characteristics of the studied patients with and without MIS-C

| **Variables** |  | | **P-value** |
| --- | --- | --- | --- |
|  | **COVID patients without MIS-C^a^** | **COVID patients with MIS-C** |  |
|  | **No. = 62** | **No. = 18** |  |
| Dyspnea, n (%) | 33 (53.2%) | 14 (77.8%) | 0.062 |
| Cardiac manifestations, n (%) | 11 (17.7%) | 17 (94.4%) | **<0.001**** |
| Hypotension, n (%) | 7 (11.3%) | 14 (77.8%) | **<0.001**** |
| Tachycardia, n (%) | 10 (16.1%) | 11 (61.1%) | **<0.001**** |
| Co-morbid conditions n (%) | 29 (46.8%) | 16 (88.9%) | **0.002**** |
| RSNA ^b^ expert consensus statement  Negative  Indeterminate  Atypical  Typical | 26 (41.9%)  23 (37.0%)  8 (12.9%)  5 (8.2%) | 6 (38.8%)  5 (27.8%)  5 (27.8%)  1 (5.6%) | 0.620 |
| CXR score  Median (IQR)  Range | 2 (0 – 4) | 3.5 (0 – 4) | 0.512 |
|  | 0 – 6 | 0 – 6 |  |
| RAPID-COVID Score  Median (IQR)  Range | 2 (1 – 6) | 4.5 (1 – 6) | 0.132 |
|  | 1 – 8 | 1 – 8 |  |
| COVID-19 severity assessment score (COSA)  Median (IQR)  Range | 3 (1 – 5) | 4 (3 – 5) | **0.014*** |
|  | 0 – 6 | 2 – 6 |  |
| Chest CT severity score (CT-SS)  Median (IQR)  Range | 6.5 (0 – 16) | 8 (0 – 14) | 0.754 |
|  | 0 – 32 | 0 – 32 |  |

^a^; Multisystem inflammatory syndrome in children, ^b^; Radiological Society of North America Expert Consensus Statement, P^*^; Significant; P^**^; Highly significant.

E-Table 2: Detailed radiological findings among the studied patients with COVID infection

| **Variables (n=80)** | **Values** |  |
| --- | --- | --- |
| Radiological findings  Bilateral peri bronchial thickenings and peri-bronchial opacities  Bilateral diffuse consolidation without specific distribution  Bilateral distribution peripheral GGOs and consolidation  Bilateral peripheral subpleural GGOs in the lower lobes  Unilateral segmental lobar consolidation  Peripheral and central GGOs  Bilateral pleural effusion  Unilateral pleural effusion  Unilateral pneumothorax  Fine atelectatic bands |  | |
|  | 9 (20%)  8(17.8%)  7(15.6)  5 (11.1%)  4 (8.9%)  3 (6.7 %)  4 (8.9%)  3 (6.7%)  3 (6.7%)  1 (2.2%) | |
|  |  |  |
| The number of lesions  Single  Multiple  Diffuse | 3 (6.7%)  16 (35.6%)  26 (57.8%) |  |
| Total opacity number, median (IQR)  Range | 1 (0 – 4) |  |
|  | 0 – 10 |  |
| Margin of the lesion  No lesion  Well defined  Ill defined | 35 (43.8%) |  |
|  | 15 (33.3%) |  |
|  | 30 (66.7%) |  |
| Largest opacity size (mm), median (IQR)  Range | 3 (0 – 5) |  |
|  | 0 – 7 |  |
| Reticular opacities | 1 (2.2%) |  |
| The affected lung sides  Bilateral  Unilateral |  |  |
|  | 36 (45.0%) |  |
|  | 9 (11.3%) |  |
| Frequency of lobe involvement Median (IQR)  Range | 2 (0 – 4)  0 – 6 |  |
| The distribution of affected lung lobes  Left lower lobe  Right lower lobe  Right middle lobe  Lingula  Right upper lobe  Left upper lobe | 33 (41.3%)  32 (40.0%)  27 (33.8%)  24 (30.0%)  9 (11.3%)  4 (5.0%) |  |
| Associated findings as Halo sign | 1 (2.2%) |  |
| Associated findings as air bronchogram | 8 (17.8%) |  |
| Associated findings as pleural thickening | 1 (2.2%) |  |
| Associated findings as interlobular interstitial thickening | 3 (6.7%) |  |
| Septal thickening | 4 (8.9%) |  |
| Bronchial wall thickening | 6 (13.3%) |  |
| Pulmonary lymphadenopathy | 3 (6.7%) |  |
| Pulmonary fibrosis | 0 (0.0%) |  |
| vascular thickening | 0 (0.0%) |  |

CT; computed tomography, GGO; ground glass opacity

E- Table 3: Correlations between COVID severity scores and the other study parameters

| **Variables** | **COVID 19 severity**  **assessment score (COSA)** | | **Chest CT severity**  **score (CT-SS)** | | **RAPID-COVID ^a^ Score** | **Chest x ray score** |
| --- | --- | --- | --- | --- | --- | --- |
|  | **r** | **P-value** | **r** | **P-value** | **r P-value** | **r P-value** |
| Age (in years) | 0.034 | 0.765 | **-.250*** | **0.025** | -0.110 0.332 | -0.050 0.65 |
| Weight(kg) | 0.037 | 0.743 | **-.237*** | **0.034** | -0.094 0.404 | -0.195 0.08 |
| Height(cm) | 0.058 | 0.609 | **-.243*** | **0.030** | -0.113 0.317 | 05. -0.221 0 |
| Body mass index kg/m2 | 0.035 | 0.758 | -.089 | 0.431 | 0.028 0.802- | 0.015 0.89 |
| Grade of fever | 0.144 | 0.203 | 0.183 | 0.104 | 0.137 0.962 | 0.17 0.152 |
| Oxygen saturation | -.149 | 0.188 | **-.274*** | **0.014** | **-.486** <0.001** | **-0.325** 0.003** |
| Respiratory rate | **.232*** | **0.038** | **.355**** | **0.001** | **.463** <0.001** | **0.411** <0.001** |
| Frequency of lobe involvement | **.406**** | **<0.001** | **.882**** | **<0.001** | **.637** <0.001** | **0.802** <0.001** |
| Chest x ray score | **.426**** | **<0.001** | **.921**** | **<0.001** | **NA** | **NA** |
| Neutrophils (10^3/ul) | **.517**** | **<0.001** | **.348**** | **0.002** | **.456** <0.001** | **0.369** <0.001** |
| Neutrophil/lymphocyte ratio | **512**** | **<0.001** | 0.215 | 0.055 | 0.316 0.113 | 0.112 0.981 |

^a^; Rapid evaluation of Anamnesis, PO2, Imaging disease, Dyspnea-Covid Score *; Significant; **: Highly significant, NA; not applicable

1. **Supplementary Figures:**

**E-Figure 1:** ROC curves to predict the cutoff values of the different COVID-19 severity scores


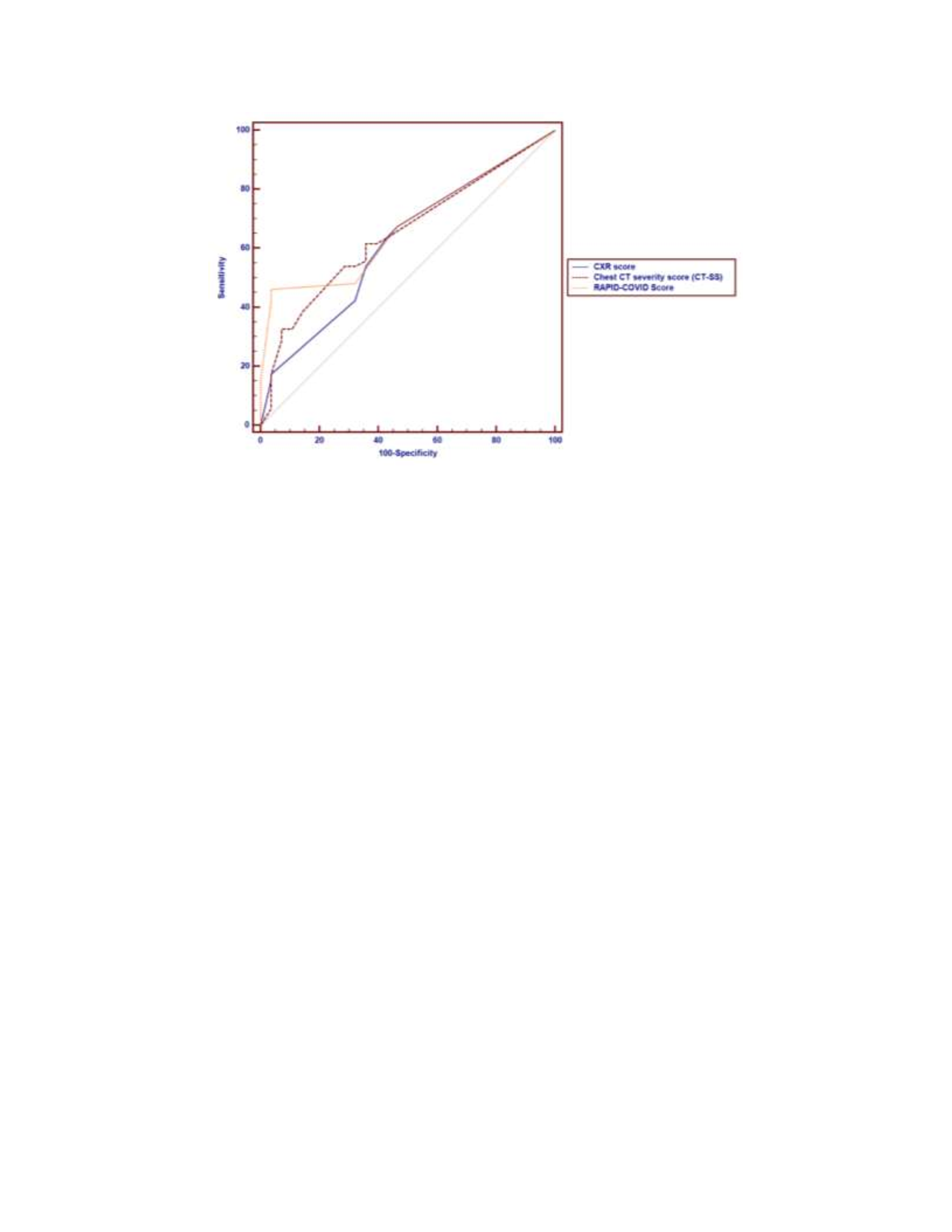


**E-Figure 2** **panel**: Relations between chest x ray score, intensive care unit (ICU) admission (ICU) (2A), mechanical ventilation (2B), complications (2C), and mortality (2D)


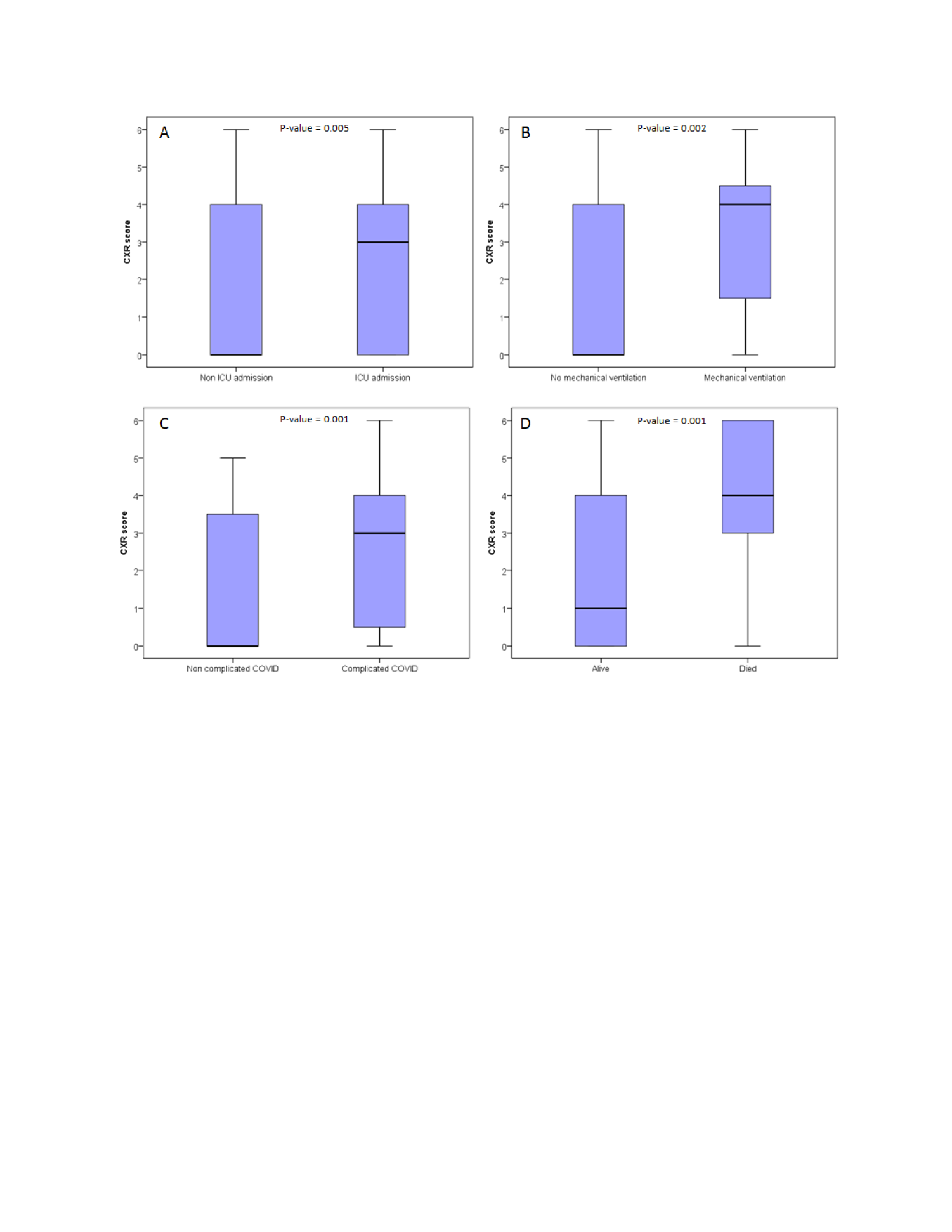


**E-Figure 3 panel**: Relations between chest CT severity score (CT-SS), intensive care unit (ICU) admission (3A), mechanical ventilation (3B), complications (3C), and mortality (3D)


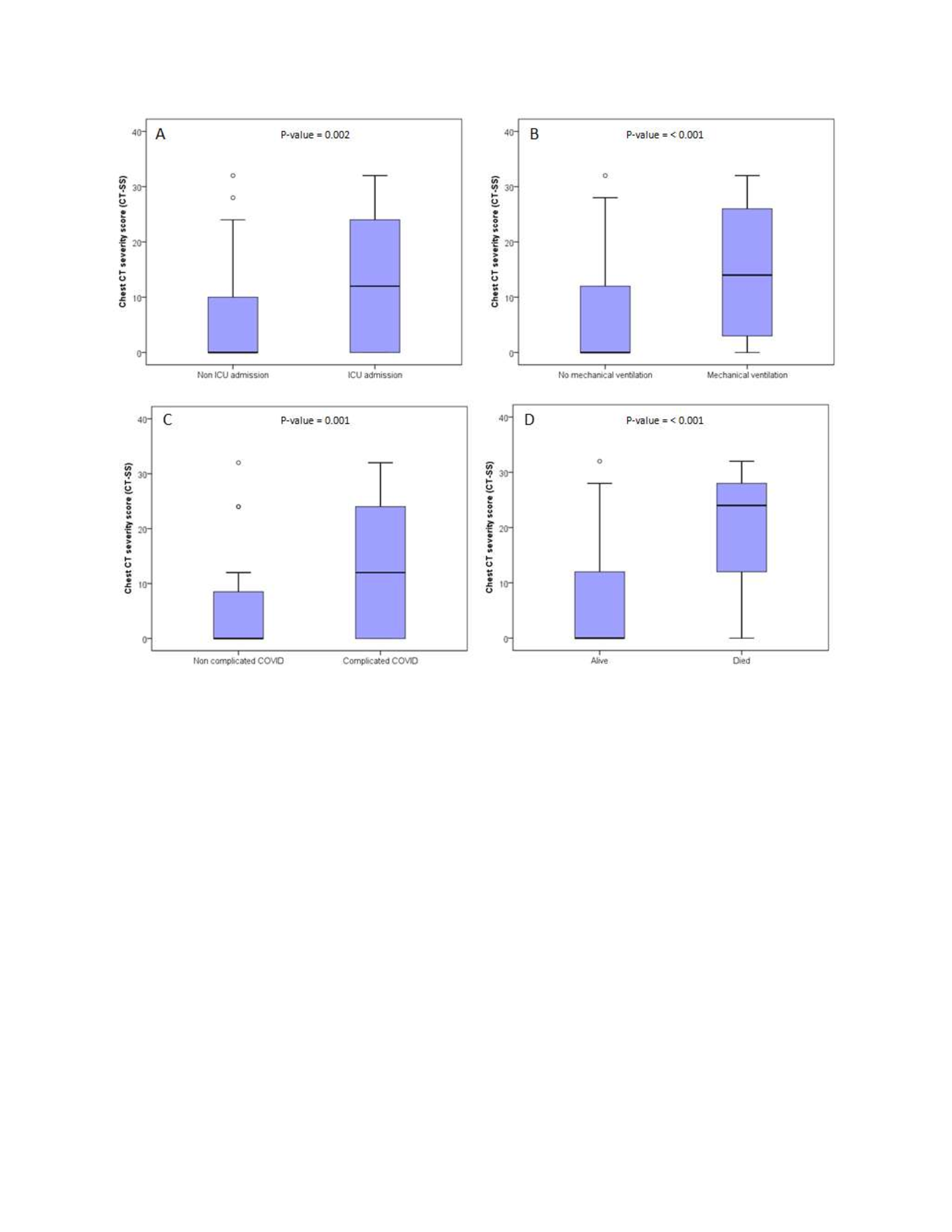

Supplement: Supplementary Materials — Supplementary data: detailed description of the severity scores used among the studied COVID-19 patients. Supplementary tables: E-Table 1: comparison between the basic characteristics of the studied patients with and without MIS-C. E-Table 2: detailed radiological findings among the studied patients with COVID-19 infection. E-Table 3: correlations between COVID-19 severity scores and the other study parameters. Supplementary figures: E-Figure 1: ROC curves to predict the cut-off values of the different COVID-19 severity scores. E-Figure 2 panel: relations between chest X-ray score, intensive care unit (ICU) admission (2A), mechanical ventilation (2B), complications (2C), and mortality (2D). E-Figure 3 panel: relations between chest CT severity score (CT-SS), intensive care unit (ICU) admission (3A), mechanical ventilation (3B), complications (3C), and mortality (3D). [file 4159651.f1.doc]
